# A Similar Metabolic Profile Between the Failing Myocardium and Tumor Could Provide Alternative Therapeutic Targets in Chemotherapy-Induced Cardiotoxicity

**DOI:** 10.3389/fcvm.2018.00061

**Published:** 2018-06-11

**Authors:** Bruno Saleme, Gopinath Sutendra

**Affiliations:** ^1^Department of Medicine, University of Alberta, Edmonton, AB, Canada; ^2^Mazankowski Alberta Heart Institute, University of Alberta, Edmonton, AB, Canada; ^3^Cancer Research Institute of Northern Alberta, University of Alberta, Edmonton, AB, Canada

**Keywords:** cardiotoxicity, cardiac metabolism, heart failure, altered metabolism, Warburg effect, cardio-oncology

Chemotherapy-induced cardiotoxicity (CIC) is an emerging clinical problem with significant healthcare costs and no preventative therapies (1, 2). Identifying selective therapeutic targets in CIC is difficult, in part, because the mechanisms of drug toxicity vary between chemotherapeutics. For example, cardiotoxicity can be acute or chronic, transient or permanent, and can affect myocardial contractility, cardiomyocyte conduction or the myocardial vascular system (3). Thus, candidate CIC therapies would need to target many features involved in cardiac dysfunction, and additionally should not prevent chemotherapy-mediated tumor regression. Although most would agree that investing in new therapies that specifically target the tumor, while not affecting other normal tissues, including the heart would be ideal, this approach is currently impractical, as even the most selective cancer therapies have been associated with cardiotoxicity (1). For example, Bcr-Abl kinase is a specific gene fusion that causes chronic myeologenous leukemia (CML) (4), and although Bcr-Abl kinase inhibitors, including imatinib mesylate are effective in treating CML (5), they are also associated with cardiotoxicity in pre-clinical animal studies and patients (6), suggesting that alternative adjuvant therapies that can prevent, limit or improve CIC need to be developed. The most commonly used preventative therapy for CIC is dexrazoxane (7), and although dexrazoxane has shown some benefit in preventing CIC (7), it has also been associated with prevention of chemotherapy-induced tumor regression (8), and increased incidence in the development of certain types of cancer in pediatric patients (9, 10). In addition, current treatment guidelines for patients diagnosed with CIC often result in discontinuation of the chemotherapy (regardless of the tumor responsiveness) and initiation into standard heart failure treatment regimes (which include β-blockers and angiotensin inhibitors) (11). In both options, for either prevention or treatment of CIC, the myocardium appears to have precedence over the tumor, with patients receiving suboptimal care for their cancer. Rather than separating our treatment regime to focus either on heart failure or cancer, an ideal approach would look for common pathways identified in both tissues, with the aim to limit or improve chemotherapy-induced heart failure, but not prevent (or even enhance) chemotherapy-induced tumor regression. In this opinion article, we will discuss metabolic pathways that appear to be induced in both the failing heart and tumor, suggesting that metabolic therapies could provide an alternative approach for treating CIC, without hindering or potentially even improving chemotherapy-induced tumor regression.

In recent years several metabolic pathways have been identified in the failing myocardium, resulting in the emergence of metabolic therapies that appear to be beneficial against several forms of heart failure in both animals and patients (12, 13). The myocardium is the most energetically demanding organ of our body, and predominantly utilizes long-chain fatty acids and glucose as the primary substrates to generate adenosine triphosphate (ATP), which is required for myocardial contractility (14). In normal conditions, glucose is metabolized to pyruvate in the cytoplasm by glycolysis (GLY), generating ~2 ATP (14). Pyruvate can be further metabolized in the mitochondria to acetyl-CoA, the substrate for the Krebs' cycle, in a process termed glucose oxidation (GO), and this requires the pyruvate dehydrogenase complex (PDC) (14). Alternatively, long-chain fatty acids can also be metabolized in the mitochondria to generate acetyl-CoA *via* fatty acid β-oxidation (FAO) (14). The reducing equivalents NADH and FADH_2_ produced from the Krebs' cycle can enter the electron transport chain (ETC) to produce ~32 ATP (14). The normal myocardium generates the majority of its ATP (~60–90%) from mitochondrial fatty acid β-oxidation (FAO) and glucose oxidation (GO), with cytoplasmic GLY providing a minimal alternative energy-producing pathway (14, 15). Several studies have reported that a transition from a normal to failing myocardium is associated with a switch in energy metabolism from mitochondrial GO to cytoplasmic GLY (13–15). Furthermore, GLY appears to be uncoupled from GO, in part, because PDC is actively inhibited by pyruvate dehydrogenase kinase (PDK) (14). The increase in GLY (and uncoupling to GO) results in an increase in the production of lactate and protons (H^+^) in the cytoplasm. This buildup of H^+^ eventually results in a decrease in cardiac efficiency, since the cardiomyocytes utilize a large amount of ATP to restore ion homeostasis, at the expense of ATP-dependent contractility (14). Thus, this shift in energy metabolism impairs cardiac contractility and conductance.

A prominent metabolic transcription factor that has been shown to be important in the switch in energy metabolism from GO to GLY is hypoxia-inducible factor 1α (HIF1α) (16). HIF1α is a transcription factor that is induced in the failing myocardium, and is associated with increased expression of glucose transporters, glycolytic enzymes, and PDK (17–19). Thus, HIF1α can directly increase GLY (*via* increasing glucose uptake into the cell and increasing the levels of glycolytic enzymes) and inhibit GO (*via* the induction of PDK), resulting in decreased cardiac efficiency. Several studies have shown that coupling GLY with GO can improve cardiac function in several heart failure models. For example, inhibition of PDK with the small molecule compound dicholoracetate (DCA) improves cardiac function in both ischemic and afterload-induced heart failure models (20–24). Furthermore, inhibition of FAO with Ranolazine or Trimetazidine, which subsequently increase GO [*via* the Randle cycle; (25)], improves cardiac function in multiple preclinical heart failure models and in patients (26–29). Therefore, increasing GO (either directly with PDK inhibitors or indirectly with FAO inhibitors) appears to reverse the metabolic remodeling observed in the failing heart and improve cardiac efficiency and function. A recent study has implicated a similar metabolic remodeling in sunitinib-induced heart failure [i.e., increased GLY (30)], suggesting that therapeutically increasing GO in CIC would be beneficial in this form of heart failure as well. In addition, several chemotherapeutics, including anthracyclines or tyrosine kinase inhibitors are associated with cardiac metabolic dysfunction (30–38), providing further evidence that metabolic therapies could be beneficial against a variety of cardiotoxic chemotherapy agents.

Intriguingly, a similar metabolic remodeling has also been identified in cancer progression (39, 40). In 1927 Otto Warburg observed that most cancer cells utilized aerobic GLY, and this was associated with decreased mitochondrial respiration (41). It is now well described that cancer cells have a similar uncoupling of GLY with GO to the failing myocardium, however, unlike the failing myocardium, this metabolic profile provides cancer cells with a survival advantage (39, 42). For example, the increase in GLY in cancer results in an increase in other glycolytic branching pathways, including the pentose phosphate pathway or serine biosynthetic pathway, which generates nucleotides or amino acids, respectively, both required for cell proliferation (40, 43). Alternatively, the decrease in mitochondrial GO provides cancer cells with apoptosis resistance (19, 39, 44). The inhibition of PDC (and GO) in cancer cells is associated with an increase in the mitochondrial membrane potential, which subsequently increases the threshold for activation of the mitochondrial permeability transition pore and thus, mitochondrial dependent apoptosis (19, 39, 44). Similar to the failing myocardium, HIF1α is also induced in cancer cells and is associated with an increase in the expression of glucose transporters, glycolytic enzymes and PDK (resulting in suppressed mitochondrial GO). Inhibition of PDK (and increasing GO), with DCA in cancer cells results in decreased proliferation and enhanced mitochondrial-dependent apoptosis, resulting in decreased tumor growth in several pre-clinical animal models (19, 44–47), and in a small clinical trial in glioblastoma patients (48). Alternatively, other compounds that also increase GO, including the pyruvate kinase activator TEPP-46, has shown benefit against tumor progression (49). Taken together, these studies provide strong evidence that therapeutically increasing GO is a valid approach for decreasing tumor progression. In addition, our group had shown that increasing GO with DCA was sufficient to decrease HIF1α activity (44), providing a strong positive feedback loop that would potentiate the increase in the GO/GLY ratio, in cancer.

Recent evidence has also implicated HIF1α with the reductive glutamine pathway in cancer (50–52). The reductive glutamine pathway is associated with decreased GO, and provides cancer cells with sufficient mitochondrial substrates (i.e., citrate) to sustain lipogenesis, a critical requirement for proliferating cancer cells (50). Similarly the reductive glutamine pathway has also been implicated in right-sided heart failure, as well (53), and inhibition of this pathway has been shown to be beneficial against both heart failure and cancer progression (51–53). Intriguingly, enhancing GO has been shown to inhibit the reductive glutamine pathway (51, 53), suggesting that metabolic therapies which increase GO could have alternative benefits against heart failure and cancer progression, in addition to altering energy metabolism.

In conclusion, a similar metabolic profile (i.e., uncoupling of GLY with GO) appears to be prominent in both heart failure and cancer (see Figure 1). Therapeutically increasing GO in either the failing myocardium or tumor results in improved cardiac function or tumor regression, respectively, suggesting that a similar metabolic therapy could be beneficial in CIC. Although intriguing, much work is required to address if metabolic therapies could be advantageous against this emerging and prominent clinical condition.

**Figure 1:**
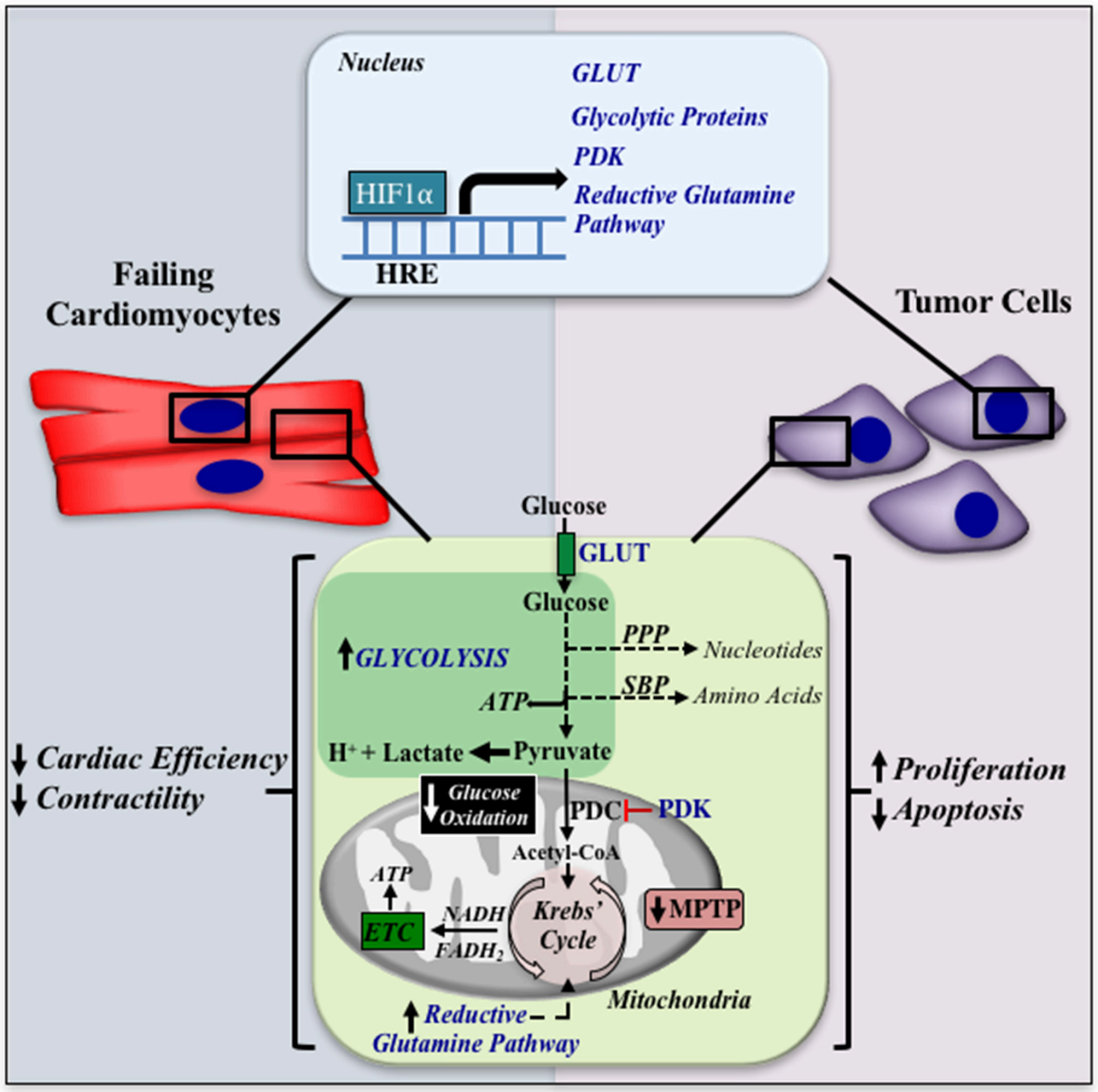
A similar metabolic remodeling in heart failure and cancer. HIF1α is induced in both the failing cardiomyocyte and cancer cells, and can increase the expression of glucose transporters, glycolytic enzymes, pyruvate dehydrogenase kinase (PDK), and the reductive glutamine pathway (shown in blue font). The increase in glycolysis is accompanied with the shuttling of glycolytic intermediates into the PPP and SBP, increasing nucleotide and amino acid synthesis, respectively. Furthermore, uncoupling of glycolysis with glucose oxidation results in an increase in lactate and H^+^ production in the cytoplasm. Inhibition of glucose oxidation is associated with closure of the MPTP, leading to apoptosis resistance. These metabolic alterations result in decreased cardiac efficiency and contractility in the heart and increased proliferation and apoptosis resistance in the tumor. HRE, Hypoxia Response Element; GLUT, Glucose Transporter; PPP, Pentose Phosphate Pathway; SBP, Serine Biosynthetic Pathway; PDK, Pyruvate Dehydrogenase Kinase; PDC, Pyruvate Dehydrogenase Complex; ETC: Electron Transport Chain; MPTP, Mitochondrial Permeability Transition Pore.

## Author contributions

All authors listed, have made substantial, direct and intellectual contribution to the work, and approved it for publication.

### Conflict of interest statement

The authors declare that the research was conducted in the absence of any commercial or financial relationships that could be construed as a potential conflict of interest. The reviewer SG and handling editor declared their shared affiliation.
